# Bone Mineral Density in Children with Cerebral Palsy: Associations with Anthropometric and Clinical Characteristics—A Cross-Sectional Study

**DOI:** 10.3390/children12070894

**Published:** 2025-07-07

**Authors:** Aqeelah Abdulelah Aljishi, Mohammed A. Al-Omari, Ayat H. Al Safar, Shahad A. AlHazzaa, Alaa I. Ibrahim

**Affiliations:** 1 Alahsa Health Cluster, Ministry of Health, Hofuf 36421, Saudi Arabia; 2 Department of Pediatrics, College of Medicine, Imam Abdulrahman Bin Faisal University, King Fahad Hospital of the University, Al-Khobar 31952, Saudi Arabia; momari@iau.edu.sa (M.A.A.-O.); ahalsafar@iau.edu.sa (A.H.A.S.); saalhazzaa@iau.edu.sa (S.A.A.); 3 Department of Physical Therapy, College of Applied Medical Sciences, Imam Abdulrahman Bin Faisal University, Dammam 31451, Saudi Arabia; aiibrahim@iau.edu.sa

**Keywords:** cerebral palsy, ambulation, bone mineral density, physical activity, spasticity

## Abstract

**Background/Objectives:** Cerebral palsy (CP) is the most common cause of neurological disability in children and is frequently associated with low bone mineral density (BMD) and increased risk of fractures. This study aimed to assess BMD in children with CP, compare it with normative standards, and explore potential associations with anthropometric parameters and the clinical characteristics of children with CP. **Methods:** Thirty-six children with CP aging 6–15 years from both sexes with varying levels of Gross Motor Functional Classification System (GMFCS) and spasticity were evaluated. Areal BMD and Z-scores (total and subtotal) were measured using dual-energy X-ray absorptiometry (DXA). Regression analysis identified predictors of BMD. **Results:** Children with GMFCS levels III–V had significantly lower total and subtotal Z-scores compared to those with levels I–II (*p* = 0.001 and *p* = 0.02, respectively). Total Z-score was significantly predicted by weight (β = 1.02, *p* = 0.002), height (β = −0.72, *p* = 0.02), and sedentary time (β = −0.47, *p* = 0.005). “No walking” was the only significant predictor for subtotal Z-score (β = −0.50, *p* = 0.004). **Conclusions:** Children with moderate to severe CP exhibited significantly lower BMD, particularly those with limited ambulation and higher spasticity levels. These findings underscore the importance of early screening and targeted interventions to optimize bone health in this population.

## 1. Introduction

Cerebral palsy (CP) is the most common cause of neurological disability in childhood, with an estimated global prevalence of 2 cases per 1000 live births [1]. It encompasses a heterogeneous group of non-progressive, permanent disorders caused by brain maldevelopment occurring in utero or during the first two years of life. These disorders result in a wide variety of abnormal postures and movements, leading to significant limitations in daily activities [2,3]. Preterm children are at particularly high risk, with an estimated incidence of 40–100 per 1000 live births, especially among those born before 28 weeks of gestation [4].

Although the brain injury in CP is static and non-progressive, its clinical manifestations often evolve over time. Primary impairments include abnormal muscle tone, loss of motor control, and muscle imbalance. These lead to secondary complications that affect musculoskeletal growth and development, particularly impairing gait and ambulation [5]. Due to reduced mobility, children with CP are susceptible to several musculoskeletal complications such as sarcopenia, muscle contractures, low bone mineral density (BMD), and fractures [6].

The reported incidence of fractures in severe forms of CP is approximately 9.7% per year [6]. Moreover, children with CP are prone to fragility fractures resulting from low-impact trauma, including routine activities such as dressing and transferring, with a prevalence of approximately 20% [7,8,9]. Most fractures affect the lower extremities, with 74% occurring in the femur [10].

The reported incidence of fractures in severe forms of CP is approximately 9.7% per year [6]. Moreover, children with CP are prone to fragility fractures resulting from low-impact trauma, including routine activities such as dressing and transferring, with a prevalence of approximately 20% [7,8,9]. Most fractures affect the lower extremities, with 74% occurring in the femur [10].

Low BMD in children with CP is multifactorial. Contributing factors include prematurity, reduced mobility [11,12], limited weight bearing [11], poor nutrition, insufficient sun exposure, use of anticonvulsants, and hormonal dysregulation—particularly involving insulin-like growth factor [13]. Muscle weakness in non-ambulant children further contributes to fracture risk by reducing mechanical loading on bones, leading to diminished periosteal bone growth and thin bones [14].

Dual-energy X-ray absorptiometry (DXA) is the recommended method for measuring BMD, as endorsed by the International Society of Clinical Densitometry. It is an accurate and valuable tool for estimating fracture risk and supporting clinical decision making [15]. The American Academy for Cerebral Palsy and Developmental Medicine recommends DXA scanning for children with CP who present with fractures and/or bone pain that may influence management decisions [16]. Due to the lack of a standardized definition of osteopenia and osteoporosis in children, the term “low bone mass” is commonly used to describe a BMD Z-score of ≤−2.0, adjusted for age, sex, and body size [17].

Although CP is the most common pediatric disorder associated with osteoporosis, studies assessing BMD and bone loss in this population remain limited, particularly in our region [18,19]. Therefore, the present study aims to assess BMD in children diagnosed with CP, compare it with normative standards, and explore its potential associations with anthropometric parameters and clinical characteristics.

## 2. Materials and Methods

### 2.1. Participants and Study Design

This cross-sectional analytical study included children diagnosed with CP who were recruited from King Fahd University Hospital and Himmah Center for Supportive Services in Al Khobar, Saudi Arabia. Participants were recruited and data were gathered during the timeframe from February to September 2022. Inclusion criteria were the following: children aged 6 to 15 years, of either sex, with any type of CP, at any level of the Gross Motor Function Classification System (GMFCS), and any degree of spasticity as measured by the Modified Ashworth Scale (MAS). Exclusion criteria included progressive neurological disorders, known bone or growth diseases, major surgery within the past 3 months, current use of casting, presence of metallic implants in the femur or lumbar spine, ongoing growth hormone therapy, or physical activity and massage interventions that promote bone mineralization. Eligible participants were evaluated by a pediatric neurologist and referred for DXA scanning. Anthropometric data were collected, including height, weight, and body mass index (BMI).

### 2.2. Clinical Characteristics

#### 2.2.1. Gross Motor Functional Classification System (GMFCS)

The GMFCS is a validated system for classifying gross motor function into five levels in children with CP [20]. It categorizes children according to their self-initiated movement capabilities, particularly sitting and walking. GMFCS levels I–II describe children who walk independently, with level I showing minimal limitations and level II involving some balance or endurance issues. Levels III–V reflect increasing mobility challenges: level III: walks with assistive devices, may use a wheelchair for long distances; levels IV–V: limited self-mobility, primarily use a wheelchair; level V includes severe motor impairments requiring full assistance. For this study, participants were grouped as having mild CP (GMFCS levels I–II) or moderate to severe CP (levels III–V).

#### 2.2.2. Muscle Tone Abnormalities

Muscle tone abnormalities were categorized as spasticity, ataxia, dyskinesia, hypotonia, and mixed. Spasticity was assessed using MAS on four muscle groups: knee extensors, ankle plantar flexors, elbow flexors, and wrist flexors. The average scores from both sides were recorded on a scale from 0 to 5 [21].

#### 2.2.3. Ambulation and Paralysis Distribution

Topographical patterns of motor involvement were categorized as monoplegia, paraplegia, hemiplegia, diplegia, quadriplegia, or double hemiplegia. Ambulation status was assessed by a physiotherapist and classified into three categories: independent ambulation (score 1), walking with assistive devices (score 2), or non-ambulant (score 3).

#### 2.2.4. Motor Characteristics

##### Isometric Muscle Strength (Handgrip)

Isometric muscle strength was measured using a Camry Digital Handgrip Dynamometer Model EH101 (Zhongshan Camry Electronic Co., Ltd., Zhongshan, China). Participants, seated comfortably, were asked to squeeze the device maximally and hold for five seconds. Three trials were performed, and the highest value (in kilograms) was recorded.

##### Physical Activities (ActiGraph)

Physical activity (PA) was assessed using the ActiGraph accelerometer (Bluetooth^®^ Smart wGT3X-BT, Kirkland, WA, USA), a validated tool for pediatric PA evaluation [22]. The device recorded activity counts, energy expenditure, wear time, and sleep parameters. Participants wore the device on their waist for four consecutive days (three weekdays and one weekend day), except during bathing. Activity intensities were categorized based on counts per minute (cpm): sedentary (<800 cpm), light (800–3200 cpm), moderate (3200–8200 cpm), and vigorous (≥8200 cpm) [23].

### 2.3. Outcome

#### 2.3.1. Measurements of Bone Mineral Density (BMD)

BMD was measured using a HOLOGIC Discovery-Ci DXA scanner (Hologic, Inc./United States (Bedford, MA, USA)). Z-scores were calculated by comparing each participant’s BMD to age- and sex-matched reference values and then dividing by the population standard deviation [24]. The total Z-score encompassed the head, trunk, arms, and legs. The subtotal Z-score represented the whole body excluding the head (WBLH).

#### 2.3.2. Assessment of Calcium Intake

Daily calcium intake was estimated using the International Osteoporosis Foundation calcium calculator (International Osteoporosis Foundation, Nyon, Switzerland). Although not formally validated, this tool was selected for its simplicity and feasibility in this population [25].

### 2.4. Statistical Analysis

Data analysis was performed using SPSS version 20.0 (SPSS Inc., Chicago, IL, USA). Continuous variables were presented as means ± standard deviations (SDs) and categorical variables as frequencies and percentages. Group comparisons were performed using independent *t*-tests or Mann–Whitney U tests, as appropriate. Paired *t*-tests were used to compare within-group differences in Z-scores and calcium intake. Pearson and Spearman correlation coefficients were used to assess relationships between BMD Z-scores and anthropometric, clinical, and motor characteristics. Stepwise multiple linear regression was used to identify significant predictors of total and subtotal Z-scores. A *p*-value < 0.05 was considered statistically significant.

## 3. Results

A total of 36 children with CP were included, with a mean age of 9.7 years (SD = 2.7). The cohort included 24 males (66.7%) and 12 females (33.3%). Based on GMFCS, 22 participants were classified as levels III and 14 as levels III–V. There were no significant differences between the two GMFCS groups in age, sex distribution, or handedness. However, children in GMFCS levels III–V had significantly lower mean weight (23.1 kg vs. 33.5 kg, *p* = 0.036), height (119.0 cm vs. 136.8 cm, *p* = 0.002), and BMI (14.6 kg/m^2^ vs. 18.4 kg/m^2^, *p* = 0.033), as shown in Table 1.

### 3.1. Clinical Characteristics

Spasticity was the predominant tone abnormality, affecting 88.9% of the cohort. Mixed tone was observed in two participants (5.6%), while ataxia and hypotonia were each seen in one participant (2.8%); no cases of dyskinesia were reported. Regarding topographical distribution, 52.8% had diplegia, 22.2% quadriplegia, and 19.4% hemiplegia, with one case each of monoplegia and paraplegia. GMFCS levels were distributed as follows: I (30.6%), II (30.6%), III (11.1%), IV (13.9%), and V (13.9%). Ambulation capacity assessment showed that 58.3% were independently ambulant, 19.4% required assistive devices, and 22.2% were non-ambulant. Significant differences were noted between children with GMFCS levels I–II CP and GMFCS levels III–V in topographical distribution (*p* = 0.008), GMFCS classification, and ambulation status (*p* < 0.001), as in Table 2.

### 3.2. Calcium Intake, Muscle Tone, and Handgrip Strength

Estimated daily calcium intake was significantly lower than the recommended intake in both groups (GMFCS I–II: *p* < 0.001; GMFCS III–V: *p* = 0.01). However, the proportion of recommended intake achieved was higher in the GMFCS III–V group (86.2%) than in the I–II group (62.0%, *p* = 0.004). Children with moderate to severe CP showed higher spasticity scores (MAS mean: 1.5 vs. 0.9, *p* = 0.001; spasticity index: 11.4 vs. 5.5, *p* < 0.001). Handgrip strength was significantly greater in children with mild CP (mean: 11.6 vs. 7.1, *p* = 0.006), as shown in Table 3.

### 3.3. Physical Activity

Children with GMFCS levels I–II demonstrated significantly higher physical activity across multiple domains, including total steps, step rate, total activity, activity rate, and the percentage of total time spent in light and moderate activities. No significant group difference was found for time spent in vigorous activity (*p* = 0.46), as shown in Table 4.

### 3.4. Dual-Energy X-Ray Absorptiometry (DXA)

Among children with moderate to severe CP (GMFCS III–V), the subtotal Z-score was significantly lower than the total Z-score (*p* = 0.04), whereas no such difference was observed in the mild CP group (GMFCS I–II, *p* = 0.80). Both total and subtotal Z-scores were significantly lower in the GMFCS III–V group compared to the GMFCS I–II group (*p* = 0.001 and *p* = 0.02, respectively). As shown in Table 5, children in the GMFCS III–V group also had significantly lower subtotal bone mineral content (BMC), subtotal BMD, total BMC, and total BMD. However, no significant differences were found in head BMC (*p* = 0.15) or head BMD (*p* = 0.51) between groups.

### 3.5. Association Between DXA Measurements and Characteristics of the Children with Cerebral Palsy

#### 3.5.1. Total Z-Score

Correlation analyses between the total Z-score and various clinical and functional variables are summarized in Table 6 and Table 7. No significant associations were found between the total Z-score and sex, hand dominance, tone type, GMFCS level, or paralysis distribution. Moderate positive correlations were identified between the total Z-score and weight (*r* = 0.39, *p* = 0.02), BMI (*r* = 0.38, *p* = 0.02), total activity (*r* = 0.34, *p* = 0.05), activity rate (*r* = 0.36, *p* = 0.04), percentage of time in light activity (*r* = 0.38, *p* = 0.04), and percentage of time in moderate activity (*r* = 0.38, *p* = 0.03). The total Z-score was negatively correlated with spasticity index (*r* = −0.37, *p* = 0.03), sedentary time (*r* = −0.43, *p* = 0.02), and ambulation capacity (*r* = −0.42, *p* = 0.01). In the stepwise regression model (Table 8), significant predictors of the total Z-score included weight (β = 1.02, *p* = 0.002), height (β = −0.72, *p* = 0.02), and sedentary time (β = −0.47, *p* = 0.005).

#### 3.5.2. Subtotal Z-Score

Correlations between the subtotal Z-score and clinical characteristics are also presented in Table 6 and Table 7. Positive correlations were observed with weight (*r* = 0.48, *p* = 0.003), height (*r* = 0.39, *p* = 0.02), BMI (*r* = 0.45, *p* = 0.006), total steps (*r* = 0.44, *p* = 0.01), step rate (*r* = 0.39, *p* = 0.03), total activity (*r* = 0.47, *p* = 0.006), activity rate (*r* = 0.43, *p* = 0.01), time in moderate activity (*r* = 0.39, *p* = 0.03), and handgrip strength (*r* = 0.36, *p* = 0.04). Negative correlations were found with MAS (*r* = −0.35, *p* = 0.03), spasticity index (*r* = −0.47, *p* = 0.004), sedentary time (*r* = −0.42, *p* = 0.02), GMFCS level (*r* = −0.45, *p* = 0.007), paralysis distribution (*r* = −0.43, *p* = 0.009), and ambulation capacity (*r* = −0.60, *p* < 0.001). Regression analysis (Table 8) identified ambulation status (“No walking”) as the only significant predictor of the subtotal Z-score (β = −0.50, *p* = 0.004).

## 4. Discussion

This cross-sectional study assessed BMD among children with CP aged 6 to 15 years with varying gross motor function and explored its associations with anthropometric, clinical, and motor characteristics. The findings revealed that both subtotal and total Z-scores were significantly lower in children with GMFCS levels III–V compared to levels I–II. This aligns with Akhter et al. [26], who reported lower lumbar spine BMD Z-scores in children with GMFCS V, and with Alvarez Zaragoza et al. [27], who also found lower BMD in quadriplegic children with severe motor impairment. Notably, unlike that study which focused on specific CP type, our study included a broader range of motor distributions.

Consistent with our results, other studies have shown lower lumbar spine BMD in children with moderate to severe CP, influenced by height, BMI, and mobility [28]. The significant negative correlation between ambulation capacity and both Z-scores further emphasizes the role of mobility. Supporting this, Chad et al. [29] and Finbråten et al. [30] found that non-ambulatory children exhibited lower Z-scores, with the inability to walk emerging as a key predictor of low BMD in the distal femur [30]. In our study, “no walking” was the strongest predictor of the subtotal Z-score, while weight, height, and sedentary time predicted the total Z-score.

These findings reinforce the importance of mechanical loading in bone modeling and remodeling. Bone tissue responds to strain from load bearing, and reduced activity limits this stimulus, disrupting bone formation and architecture [7]. Thus, regular physical loading remains essential for maintaining skeletal integrity, a challenge for many children with CP [31].

Interestingly, no association was observed between calcium intake and BMD Z-scores. Similar findings were reported by Unay et al. [32], while Henderson et al. [33] observed a relationship between serum calcium and BMD that diminished after adjusting for severity and nutrition. This suggests that bone health in CP is multifactorial, influenced not only by calcium intake but also by hormonal, mechanical, and nutritional factors [34].

Muscle strength, assessed via handgrip, was significantly higher in children with GMFCS I–II and positively correlated with the subtotal Z-score. This supports previous research by Chen et al. [35], who reported that greater muscle strength, particularly knee extensors, was associated with higher BMD. These results highlight the role of muscle function in skeletal development.

Physical activity was significantly higher in the GMFCS I–II group across most parameters, except for vigorous activity, where no difference was found. This is consistent with a study showing similar levels of vigorous activity across CP severity levels and greater moderate activity in children with less severe CP [36]. The discrepancy in sedentary time between studies may relate to contextual and environmental factors such as access to recreational facilities and family engagement.

To our knowledge, few studies have examined the subtotal Z-score in CP children. In our study, subtotal Z-scores were notably lower in children with more severe CP, even more so than total Z-scores. One explanation may be that head BMD remains relatively unaffected by motor severity, while limb bones, subject to less weight bearing in severe cases, are more vulnerable to demineralization.

These findings are consistent with a systematic review that reported mean Z-scores ranging from −3.4 in the distal femur to −0.8 in the lumbar spine, with 77% of children with severe CP presenting Z-scores below −2 [37]. Recognizing this risk supports the need for early screening and intervention.

Study limitations include its cross-sectional design, which precludes causality, and the small sample size, limiting generalizability. Physical activity was monitored for only four days, potentially underrepresenting habitual activity levels. Additionally, the IOF calcium calculator has not undergone formal validation in an academic context and may not encompass all dietary sources, which restricts its accuracy. Future research should aim for larger cohorts and longer monitoring durations to better characterize BMD trends in this population. Larger groups may enable stratified analysis concerning anticonvulsant therapy, in addition to examining the relationship between the effective incidence of fractures within the studied population and their clinical and anthropometric traits.

## 5. Conclusions

Our study evaluated BMD in children with various types of CP and examined its association with anthropometric, clinical, and motor characteristics. A marked decrease in BMD was observed in children with moderate to severe CP. BMD was positively correlated with weight, BMI, physical activity, handgrip strength, and ambulation capacity. Regression analysis identified weight, height, and sedentary time as significant predictors of the total Z-score, while “no walking” was the sole predictor of the subtotal Z-score. Children with moderate to severe CP are at increased risk for low BMD, particularly those who are non-ambulatory and have poor muscle strength. Early identification and targeted interventions including early physical activity programs conducted by qualified caregivers and therapists, adequate nutrition, and, when appropriate, pharmacological treatment may help optimize bone health and reduce the risk of fractures.

## Figures and Tables

**Table 1:** Demographic and anthropometric characteristics of children with CP.

Characteristics			All Children	GMFCS I–II	GMFCS III–V	*p*-Value	95% Confidence Interval	
							Lower	Upper
Number			36	22	14			
Age (years)	Mean (SD)		9.7 (2.7)	10.4 (2.5)	8.7 (2.9)	0.077	−0.19	3.49
Gender	Male	*n*	24	15	9	0.800		
		%	66.7	68.2	64.3			
	Female	*n*	12	7	5			
		%	33.3	31.8	35.7			
Handedness	Right	*n*	30	18	12	0.800		
		%	83.3	81.8	85.7			
	Left	*n*	6	4	2			
		%	16.7	18.2	14.3			
Weight (kg)	Mean (SD)		29.4 (14.8)	33.5 (12.6)	23.1 (16.0)	0.036	0.70	20.19
Height (cm)	Mean (SD)		129.9 (17.5)	136.8 (13.6)	119.0 (17.8)	0.002	7.14	28.49
BMI (kg/m^2^)	Mean (SD)		16.9 (5.2)	18.4 (5.0)	14.6 (4.9)	0.033	0.31	7.18

GMFCS: Gross Motor Functional Classification System.

**Table 2:** Clinical characteristics of children with CP (Mann–Whitney test).

Characteristics		All Children	GMFCS I–II	GMFCS III–V	*p*-Value
*n* (%)					
Number		36 (100)	22 (61.1)	14 (38.9)	
Tone Abnormality, *n* (%)	Spasticity	32 (88.9)	18 (81.8)	14 (100)	0.1
	Dyskinesia	None	None	None	
	Ataxia	1 (2.8)	1 (4.5)	None	
	Hypotonia	1 (2.8)	1 (4.5)	None	
	Mixed	2 (5.6)	2 (9.1)	None	
Distribution of Paralysis, *n* (%)	Monoplegia	1 (2.8)	1 (4.5)	None	0.008
	Paraplegia	1 (2.8)	1 (4.5)	None	
	Hemiplegia	7 (19.4)	7 (31.8)	None	
	Diplegia	19 (52.8)	10 (45.5)	9 (64.3)	
	Quadriplegia	8 (22.2)	3 (13.6)	5 (35.7)	
GMFCS, *n* (%)	I	11 (30.6)	11 (50)	None	<0.001
	II	11 (30.6)	11 (50)	None	
	III	4 (11.1)	None	4 (28.6)	
	IV	5 (13.9)	None	5 (35.7)	
	V	5 (13.9)	None	5 (35.7)	
Ambulation Capacity	Walks independently	21 (58.3)	20 (90.9)	1 (7.1)	<0.001
	Walks with a mobility device	7 (19.4)	2 (9.1)	5 (35.7)	
	No walking	8 (22.2)	None	8 (57.1)	

GMFCS: Gross Motor Functional Classification System.

**Table 3:** Muscle tone, handgrip strength, and calcium intake in children with CP (independent *t*-test).

Characteristics		All Children		GMFCS I–II		GMFCS III–V		*p*-Value	95% Confidence Interval	
									Lower	Upper
Muscle Tone, Mean (SD)	Modified Ashworth Scale	1.1 (0.5)		0.9 (0.4)		1.5 (0.6)		0.001	−0.9	−0.2
	Spasticity Index	7.8 (5.1)		5.5 (3.0)		11.4 (5.5)		<0.001	−8.9	−3.1
Hand Grip Strength in Newton, Mean (SD)		10.0 (4.8)		11.6 (4.2)		7.1 (4.6)		0.006	1.4	7.6
Estimated Daily Calcium Intake in mg *, Mean (SD)		825.1 (276.6)	<0.001 *	751.0 (306.7)	<0.001 *	941.5 (173.4)	0.01 *	0.04	−373.8	−7.2
Recommended Daily Calcium Intake in mg *, Mean (SD)		1175.0 (150.0)		1218.2 (136.8)		1107.1 (149.2)		0.03	12.6	209.4
Percentage of Estimated Daily Calcium Intake, Mean (SD)		71.4 (25.6)		62.0 (25.2)		86.2 (18.7)		0.004	−40.1	−8.2

GMFCS: Gross Motor Functional Classification System; * paired sample *t*-test for within-group analyses.

**Table 4:** Measurements of physical activities in children with CP (independent *t*-test).

Parameters	All Children	GMFCS I–II	GMFCS III–V	*p*-Value	95% Confidence Interval	
Mean (SD)					Lower	Upper
Total Steps (count)	20,537.7 (12,837.5)	27,186.4 (11,327.2)	9456.6 (5338.1)	˂0.001	10,588.3	24,871.3
Step Rate (step/min)	5.6 (3.0)	7.1 (2.7)	3.1 (1.4)	˂0.001	2.3	5.8
Total Activity (count)	3,930,627.1 (2,126,078.3)	4,878,730.9 (2,002,750.9)	2,350,454.1 (1,205,136.5)	˂0.001	1,221,044.4	3,835,509.2
Activity Rate (activity/min)	1083.1 (476.3)	1273.4 (435.8)	766.9 (369.3)	0.002	198.8	814.2
Percentage of Total Sedentary Time (%)	66.3 (11.0)	62.1 (9.5)	73.5 (9.9)	0.003	−18.6	−4.2
Percentage of Total Time in Light Activities (%)	15.5 (4.1)	16.7 (3.9)	13.5 (3.8)	0.03	0.3	6.0
Percentage of Total Time in Moderate Activities (%)	18.0 (8.3)	21.1 (7.2)	12.8 (7.6)	0.005	2.7	13.7
Percentage of Total Time in Vigorous Activities (%)	0.2 (0.2)	0.2 (0.2)	0.1 (0.2)	0.46	−0.1	0.2

GMFCS: Gross Motor Functional Classification System.

**Table 5:** Measurements of dual-energy X-ray absorptiometry in children with CP (independent *t*-test).

Parameters	All Children		GMFCS I–II		GMFCS III–V		*p*-Value	95% Confidence Interval	
Mean (SD)								Lower	Upper
Subtotal BMC (g)	610.8 (294.3)		699.1 (290.4)		472.14 (251.18)		0.02	35.1	418.8
Subtotal BMD (g/cm^2^)	0.60 (0.14)		0.66 (0.12)		0.51 (0.12)		0.001	0.06	0.2
Head BMC (g)	265.51 (49.63)		275.14 (52.48)		250.37 (42.19)		0.15	−9.1	58.7
Head BMD (g/cm^2^)	1.31 (0.17)		1.32 (0.19)		1.28 (0.14)		0.51	−0.1	0.2
Total BMC (g)	907.7 (322.2)		1025.5 (296.5)		722.6 (277.5)		0.004	101.8	504.0
Total BMD (g/cm^2^)	0.72 (0.11)		0.76 (0.11)		0.66 (0.10)		0.005	0.03	0.2
Subtotal Z-Score *	−2.29 (1.35)	0.1 *	−1.74 (1.01)	0.8 *	−3.17 (1.40)	0.04 *	0.02	0.1	1.6
Total Z-Score *	−2.09 (1.09)		−1.76 (0.95)		−2.61 (1.13)		0.001	0.6	2.2

GMFCS: Gross Motor Functional Classification System; BMC: bone mineral content; * paired sample *t*-test for within-group analyses.

**Table 6:** Correlations between DXA measurements and characteristics of the children with CP (Pearson correlation).

Independent Variables	Total Z-Score		Subtotal Z-Score	
	r	*p*	r	*p*
Age	0.13	0.45	0.17	0.31
Weight	0.39	0.02	0.48	0.003
Height	0.23	0.19	0.39	0.02
BMI	0.38	0.02	0.45	0.006
Average Modified Ashworth Scale	−0.30	0.07	−0.35	0.03
Spasticity Index	−0.37	0.03	−0.47	0.004
Total Steps	0.33	0.07	0.44	0.01
Step Rate	0.33	0.07	0.39	0.03
Total Activity	0.34	0.05	0.47	0.006
Activity Rate	0.36	0.04	0.43	0.01
Percentage of Total Sedentary Time	−0.43	0.02	−0.42	0.02
Percentage of Total Time in Light Activities	0.38	0.04	0.34	0.06
Percentage of Total Time in Moderate Activities	0.38	0.03	0.39	0.03
Percentage of Total Time in Vigorous Activities	0.06	0.74	0.14	0.45
Hand Grip Strength	0.26	0.13	0.36	0.04
Percentage of Estimated Daily Calcium Intake	−0.12	0.50	−0.20	0.27

DXA: Dual-energy X-ray absorptiometry; r: correlation coefficient; *p*: significant correlation.

**Table 7:** Correlations between DXA measurements and characteristics of the children with CP (Spearman correlation).

Independent Variables	Total Z-Score		Subtotal Z-Score	
	r	*p*	r	*p*
Gender	−0.08	0.65	0.04	0.83
Hand Dominance	0.25	0.15	0.11	0.53
Type of Tonal Abnormality	−0.09	0.61	−0.02	0.91
Gross Motor Functional Classification System	−0.29	0.09	−0.45	0.007
Distribution of Paralysis	−0.29	0.09	−0.43	0.009
Ambulation Capacity	−0.42	0.01	−0.60	˂0.001

DXA: Dual-energy X-ray absorptiometry; r: correlation coefficient; *p*: significant correlation.

**Table 8:** Predicting DXA total and subtotal Z- scores in children with CP (multiple linear regression analysis).

Independent Variables	Total Z-Score								
	B	SEB	β	t	Sig	R	R^2^	Adjusted R^2^	Durbin Watson
Model Summary	4.13	2.14	-	1.93	0.06	0.63	0.40	0.33	2.18
Weight	0.07	0.02	1.02	3.37	0.002	-	-	-	-
Percentage of Total Sedentary Time	−0.05	0.02	−0.47	−3.04	0.005	-	-	-	-
Height	−0.04	0.02	−0.72	−2.40	0.02	-	-	-	-
	Subtotal Z-Score								
Model Summary	−1.9	0.21	-	−8.94	˂0.001	0.50	0.25	0.22	1.81
No Walking	−1.50	0.48	−0.50	−3.11	0.004				

B: unstandardized coefficients; SEB: Std. Error of B; β: standardized coefficients; R: multiple correlation coefficients.

## Data Availability

The data presented are available upon request from the corresponding author.

## Abbreviations

The following abbreviations are used in this manuscript:

BMD	Bone Mineral Density
BMC	Bone Mineral Content
BMI	Body Mass Index
CP	Cerebral Palsy
DXA	Dual-Energy X-ray Absorptiometry
GMFCS	Gross Motor Functional Classification System
PA	Physical Activity
WBLH	Whole Body Less Head
